# Pharmacological and Pharmacokinetic Profile of Cannabidiol in Human Epilepsy: A Review of Metabolism, Therapeutic Drug Monitoring, and Interactions with Antiseizure Medications

**DOI:** 10.3390/biom15121668

**Published:** 2025-11-30

**Authors:** Ji-Hoon Na, Young-Mock Lee

**Affiliations:** Departments of Pediatrics, Gangnam Severance Hospital, Yonsei University College of Medicine, Seoul 135-720, Republic of Korea; jhnamd83@yuhs.ac

**Keywords:** cannabidiol, metabolism, pharmacokinetics, therapeutic drug monitoring, drug–drug interactions

## Abstract

Cannabidiol (CBD) has transitioned from anecdotal use to an evidence-based adjunctive therapy for Lennox–Gastaut syndrome, Dravet syndrome, and tuberous sclerosis complex. This review integrates knowledge on CBD’s pharmacology, pharmacokinetics, and clinical implementation, with focus on metabolism, therapeutic drug monitoring (TDM), and clinically relevant interactions with antiseizure medications. CBD exerts CB1/CB2-independent mechanisms—prominently GPR55 antagonism, TRP-channel desensitization, and adenosine-mediated network dampening—supporting efficacy across heterogeneous seizure phenotypes. Its pharmacokinetic profile is characterized by low and variable oral bioavailability, a pronounced food effect, extensive tissue distribution, and phase I/II biotransformation to the active 7-hydroxy-CBD and abundant 7-carboxy-CBD, resulting in substantial inter-individual variability and liability for drug–drug interactions. Clinically salient interactions include CYP2C19-mediated elevation of N-desmethylclobazam and increased transaminases in valproate co-therapy. We summarize emerging TDM practices—standardized fed-state trough sampling with paired measurement of CBD and 7-hydroxy-CBD—and discuss how preliminary interpretive ranges can support dose optimization, adherence assessment, and safety surveillance. Practical recommendations emphasize interaction-aware titration within evidence-based dose bands, liver function monitoring, and standardized documentation of formulation and sampling conditions. Future work should align pharmacogenomics with TDM, refine bioavailability through advanced delivery systems, and tighten analytical and product-quality standards to consolidate CBD as a precision-ready component of modern epilepsy care.

## 1. Introduction

Epilepsy is one of the most common serious neurological disorders. A substantial proportion of patients, often cited as about one-third, develop drug-resistant epilepsy despite adequate trials of appropriate antiseizure medications (ASMs), and these individuals experience a disproportionate burden of cognitive impairment, psychiatric comorbidities, social stigma, and reduced quality of life [1]. Drug-resistant epilepsy is also associated with increased risks of injury, hospitalization, and premature mortality, including sudden unexpected death in epilepsy. Against this background, interest in novel treatment options such as cannabidiol (CBD) has grown, particularly for severe developmental and epileptic encephalopathies in which conventional therapies often fail [1,2,3,4]. 

CBD, a non-intoxicating phytocannabinoid from *Cannabis sativa*, has advanced from historical, anecdotal use to an evidence-based therapeutic option in developmental epileptic encephalopathies [1,2,3,4]. Pivotal randomized controlled trials and subsequent regulatory approvals in the United States and Europe have established highly purified, plant-derived CBD as an adjunctive treatment for Lennox–Gastaut syndrome (LGS), Dravet syndrome (DS), and tuberous sclerosis complex (TSC). These trials demonstrated clinically meaningful reductions in seizure frequency alongside a tolerable safety profile [5,6,7,8]. These advances have solidified CBD’s role in contemporary epilepsy management while underscoring the need to delineate its mechanistic actions and optimal clinical use.

The renewed scientific focus on CBD reflects a broader trend toward non-psychoactive cannabinoids as potential antiseizure agents. CBD exerts anticonvulsant effects via a diverse, multi-target pharmacology that extends beyond classical endocannabinoid receptors, involving targets such as GPR55, transient receptor potential (TRP) channels, 5-HT1A receptors, adenosinergic signaling, and other neuronal targets implicated in excitability and neural network stability [9,10,11]. This pleiotropy is supported by convergent preclinical and translational data and provides a mechanistic rationale for efficacy across heterogeneous epileptic syndromes [12,13].

Despite its therapeutic promise, clinical implementation is complicated by pharmacokinetic (PK) challenges: notably low and variable oral bioavailability, extensive first-pass metabolism, high plasma protein binding, and substantial inter- and intra-individual variability [2,13,14,15]. CBD undergoes biotransformation primarily via cytochrome P450 enzymes CYP2C19 and CYP3A pathways, as well as glucuronidation, producing active metabolites including 7-hydroxy-CBD and downstream 7-carboxy-CBD. These metabolic pathways predispose CBD to significant drug–drug interactions within the polytherapy context typical of refractory epilepsy [2,13,16,17,18]. Clinically salient interactions include reciprocal modulation with clobazam via CYP2C19 inhibition and reports of hepatic enzyme elevations that warrant monitoring, particularly when combined with other hepatically metabolized ASMs [4,7,12].

Given that approximately one-third of people with epilepsy remain drug-resistant, precision strategies to individualize CBD therapy are essential [11,16,19]. Recent therapeutic drug monitoring (TDM) studies in real-world cohorts document wide PK variability, define preliminary reference ranged for CBD and 7-hydroxy-CBD, and demonstrate relationships among dosing, drug exposure, and biochemical markers. Collectively, these findings support TDM as a pragmatic tool for dose optimization and enhancing safety surveillance [2]. Building on this foundation, this review synthesizes the knowledge on CBD’s pharmacology, PK and metabolic pathways, and clinical trial evidence in LGS, DS, and TSC; outlines the rationale and methodologies underpinning TDM; and comprehensively reviews clinically relevant interactions with ASMs [19,20,21]. Our goal is to provide clinicians and researchers with clear, evidence-based guidance for the safe and integration of CBD into refractory epilepsy treatment paradigms, while identifying the key gaps to inform future research directions.

## 2. Pharmacological Properties of Cannabidiol

### 2.1. Molecular Targets and Mechanisms

CBD is best conceptualized as a pleiotropic neuromodulator whose antiseizure activity arises largely independent of CB1/CB2 receptor agonism, thereby avoiding classical cannabinoid psychotropic effects [10,20]. Mechanistic evidence supports antagonism of GPR55 signaling within excitatory circuits, a pathway linked to reduced intracellular Ca^2+^ release and attenuation of network hyperexcitability [22]. CBD also desensitizes TRPV1 channels, limiting Ca^2+^ influx during repetitive activity and thereby reducing activity-dependent neuronal firing. An adenosinergic component is implicated through the inhibition of equilibrative nucleoside transporter 1 (ENT1), which elevates extracellular adenosine concentration and engages A1 receptor-mediated anti-excitatory effects [9,22]. At the serotonergic level, partial agonism or positive modulation at 5-HT1A receptors has been described and may influence anxiety, arousal, and seizure thresholds in developmental epileptic encephalopathies [23]. Additional modulatory actions—on voltage-gated sodium (Na^+^) and potassium (K^+^) channels, T-type (Ca^2+^) channels, glycine receptors, and the voltage-dependent anion channel (VDAC)—likely cooperate to yield a distributed, redundancy-rich anticonvulsant profile [22]. Beyond membrane excitability, CBD exerts anti-inflammatory and antioxidant effects that mitigate seizure-driven neuroimmune cascades and oxidative stress, processes increasingly recognized as seizure amplifiers [9,24]. Emerging metabolic data suggest CBD can support mitochondrial function and cellular bioenergetics under metabolic stress, offering an additional neuroprotective axis with potential relevance to epileptogenesis [25]. CBD’s high lipophilicity, variable intestinal absorption, and extensive first-pass metabolism (notably via CYP2C19 and CYP3A4) set the pharmacologic context in which these mechanisms operate and predict clinically meaningful drug–drug interactions [22,26,27].

### 2.2. Preclinical and Clinical Evidence

Across established rodent seizure paradigms—including maximal electroshock, pentylenetetrazol, 6 Hz psychomotor, kindling, audiogenic, and genetic models—CBD consistently lowers seizure susceptibility and severity without causing motor toxicity, indicating a true antiseizure effect rather than a model-bounded artifact [9]. Mechanistic readouts from these preparations align with GPR55 antagonism, TRP-channel desensitization, and adenosine-mediated inhibition, with resultant decreases in intracellular calcium flux, reduced excitatory transmission, and stabilization of hyperexcitable networks. CBD has recently been shown to interact with the N-terminal domain of AMPARs (GluA1/GluA2 complex), leading to receptor inhibition and a reduction in neuronal excitability. This suggests a novel target for CBD’s anticonvulsant effects. Additionally, CBD modulates both excitatory glutamate and inhibitory GABA transmission. It facilitates glutamate release through TRPV1 receptor activation and enhances GABAergic transmission via GPR55 antagonism, particularly in the basal ganglia. Interestingly, CBD’s effects on neurotransmission may vary with concentration: at low concentrations, it inhibits excitability, whereas at higher concentrations, it increases glutamate release. Furthermore, CBD blocks T-type calcium channels, producing a hyperpolarizing shift in neuronal activation, which helps inhibit synchronized depolarization typical of generalized seizures, an action not shared by many other antiseizure medications [9,10,13].

The bench-to-bedside trajectory was first validated in DS, where randomized, double-blind, placebo-controlled trials of adjunctive, highly purified, plant-derived CBD showed significant reductions in monthly convulsive-seizure frequency versus placebo and higher 50% responder rates; open-label extensions documented maintenance of effect over many months with broadly consistent safety profiles [10,28]. In LGS, two phase-3 programs demonstrated clinically meaningful decreases in atonic (drop) seizures at 10 and 20 mg/kg/day, together with improvements in non-drop seizure types and patient- or caregiver-reported global impression, establishing class-I evidence for adjunctive use [1]. In TSC, a randomized phase-3 study confirmed reductions in overall seizure burden and supported regulatory label expansion to this genetically defined epileptic encephalopathy [29]. Across these syndromes, adverse events have been predictable and largely dose-related—most commonly somnolence, decreased appetite, and diarrhea—with transaminase elevations occurring more frequently in patients receiving concomitant valproate, underscoring the need for laboratory surveillance during titration [30]. Pharmacokinetic-pharmacodynamic factors are integral to interpreting trial outcomes: exposure varies widely with formulation, food effects, hepatic function, and background ASMs, and the interaction with clobazam (mediated by increased N-desmethylclobazam) can amplify both antiseizure efficacy and sedation, often necessitating dose adjustments [31,32]. Dose-finding studies and systematic dosing reviews converge on 10–20 mg/kg/day as a commonly effective adjunctive range in these disorders, while real-world therapeutic drug monitoring increasingly documents broad between-patient dispersion in CBD and active-metabolite concentrations, motivating individualized titration and periodic biochemical safety checks to balance seizure control with tolerability [9].

## 3. Pharmacokinetics of Cannabidiol

### 3.1. Absorption and Bioavailability

CBD exhibits low and highly variable oral bioavailability, commonly approximating ~6% because of extensive first—pass hepatic metabolism and solubility—limited intestinal uptake. The liberation of CBD primarily depends on the formulation and its release characteristics, which can influence its absorption rate [33]. Systemic CBD exposure rises markedly with food; high-fat meals increase AUC several-fold, underscoring the need to standardize administration conditions in clinical practice [33,34]. Time to peak concentration (Tmax) after oral dosing typically occurs within 1–2 h, but intersubject variability is large and reflects both gastrointestinal transit and different metabolic sources [33]. Route of administration and formulation materially influence exposure: relative bioavailability differs among oral oils, oromucosal sprays, and engineered lipid/nanoparticle formulations, exemplified by PTL401 soft-gel capsules that enhanced systemic availability compared with oromucosal delivery [33]. Given CBD’s pronounced food effect and formulation dependence, consistent fed-state dosing and attention to excipient systems are integral to reduce PK variability during therapy [33,35].

### 3.2. Distribution

After absorption, CBD distributes extensively into tissues, consistent with very high plasma protein binding (≈94–99%) and a remarkably large apparent volume of distribution (≈20,000–40,000 L), properties that prolong the terminal elimination half-life and predispose to displacement interactions [9,33]. CBD is a lipophilic compound and exhibits extensive distribution into various tissues, including the liver, muscle, and central nervous system (CNS). In rats, CBD has been observed to concentrate in the liver shortly after administration. Its ability to cross the blood–brain barrier and accumulate in the CNS is essential for its anticonvulsant activity, whereas the high bound fraction means small changes in albumin concentration or competitive binding at transport proteins can disproportionately alter the unbound (pharmacologically active) fraction [33]. Earlier reviews emphasized CBD’s peripheral and central actions attributable to its physicochemical profile, aligning with contemporary PK observations of deep tissue distribution [36].

### 3.3. Metabolism

CBD clearance is dominated by oxidative biotransformation via CYP3A4 and CYP2C19 to the active 7-hydroxy-CBD (7-OH-CBD), followed by further oxidation to the abundant, largely inactive 7-carboxy-CBD (7-COOH-CBD); downstream conjugation is mediated mainly by UGT1A9 and UGT2B7 [33,37]. CBD is metabolized into 7-OH-CBD as one of its primary metabolites, though only a portion of CBD undergoes this transformation. The percentage of CBD converted into 7-OH-CBD varies depending on the individual’s metabolic rate and genetic factors, particularly polymorphisms in the CYP2C19 enzyme [11,37]. CYP modulation produces clinically relevant exposure shifts: rifampin-like inducers can decrease CBD concentrations, whereas azole antifungals (e.g., fluconazole, itraconazole) increase them—effects reflected in controlled interaction studies and summarized in antiseizure-drug interaction guidance [37]. In healthy-volunteer trials designed to reflect epilepsy polytherapy, CBD showed bidirectional interactions with clobazam, stiripentol, and valproate—raising N-desmethylclobazam via CYP2C19 inhibition and showing metabolite shifts with stiripentol—evidence that motivates proactive monitoring when CBD is added to complex regimens. Mechanistic reviews and earlier summaries converge on this pathway map, reinforcing that 7-OH-CBD contributes to antiseizure activity while 7-COOH-CBD predominates in circulation. Taken together, these data indicate that CBD has a pharmacokinetic profile characterized by slow oral absorption with food-dependent bioavailability, extensive distribution into peripheral tissues and the brain, high plasma protein binding, and almost complete hepatic metabolism via CYP2C19, CYP3A, and UGT. Terminal half-life estimates typically range from approximately two to five days, and steady-state concentrations are reached only after one to two weeks of repeated dosing. Exposure increases more than proportionally with dose at the upper end of the approved range, and substantial inter-individual variability is observed, driven by factors such as body composition, hepatic function, concomitant medications, and feeding state [24,33,36].

### 3.4. Excretion

**Figure 1:**
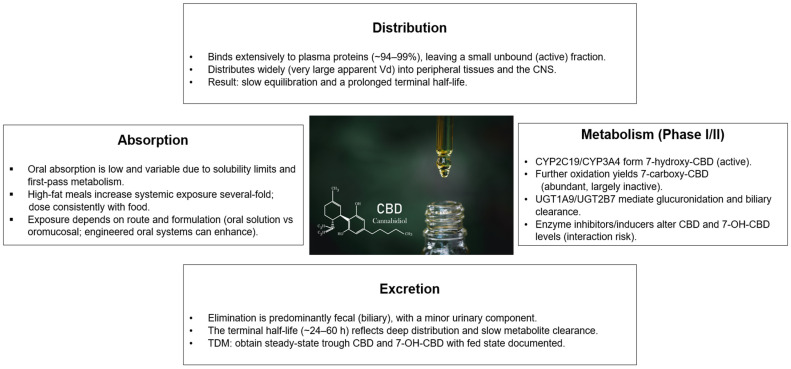
Cannabidiol Pharmacokinetics: Absorption, Distribution, Metabolism, and Excretion.

Elimination is predominantly fecal, consistent with biliary excretion of oxidized and glucuronidated metabolites, and only a minor urinary component (≈12% unchanged) is reported, aligning with CBD’s lipophilicity and extensive metabolic clearance. The long terminal half-life (≈24–60 h) reflects deep tissue distribution and slow elimination of conjugated metabolites, implications that affect accumulation and washout considerations during therapeutic drug monitoring [26,33] (Figure 1).

Schematic overview of the Absorption–Distribution–Metabolism–Excretion profile of CBD. Oral absorption is low and variable due to solubility limits and first-pass metabolism; high-fat meals increase systemic exposure, so consistent fed-state dosing is recommended. CBD exhibits high plasma protein binding and an exceptionally large apparent volume of distribution with CNS penetration, contributing to a prolonged terminal half-life. Phase I metabolism via CYP2C19/CYP3A4 generates the active 7-hydroxy-CBD, followed by formation of 7-carboxy-CBD and Phase II glucuronidation mainly by UGT1A9/UGT2B7; elimination is predominantly fecal with a minor urinary component. For therapeutic drug monitoring, trough concentrations of CBD and 7-OH-CBD at steady state should be interpreted with feeding state and formulation documented.

Abbreviations: CBD, cannabidiol; CNS, central nervous system; Vd, volume of distribution; t½, terminal half-life; CYP, cytochrome P450; UGT, UDP-glucuronosyltransferase; 7-OH-CBD, 7-hydroxy-cannabidiol; 7-COOH-CBD, 7-carboxy-cannabidiol; TDM, therapeutic drug monitoring.

### 3.5. Inter-Individual Variability

Inter-individual variability in CBD exposure is substantial and stems from differences in absorption (food, formulation), protein binding, and metabolic capacity—including variability in CYP3A4/CYP2C19 activity and UGT pathways—producing wide ranges in C_max_ and AUC under routine conditions. Meal composition studies and clinical observations consistently show fed-state dosing reduces within-subject fluctuation and improves predictability, supporting label-consistent co-administration with food [34,35]. Concomitant ASMs further modulate exposure: enzyme inducers lower CBD concentrations, whereas inhibitors raise them; in parallel, CBD can inhibit CYP2C19 and elevate N-desmethylclobazam concentrations, necessitating reciprocal dose adjustments and laboratory surveillance [37,38]. Age-dependent pharmacology and constraints of pediatric formulation add additional layers of variability, reinforcing the need for realistic dose titration and attention to developmental PK principles. Finally, the practical implementation of therapeutic drug monitoring is facilitated by validated UHPLC-MS/MS assays and is increasingly advocated to navigate the combined effects of food, genetics, hepatic function, and polytherapy on steady-state exposure [35].

## 4. Therapeutic Drug Monitoring of Cannabidiol: Integration of Metabolic Pathways with Clinical Practice

### 4.1. Understanding CBD Metabolism for TDM

CBD is primarily metabolized through oxidative pathways involving CYP2C19 and CYP3A4. These enzymes convert CBD to the active metabolite 7-hydroxy-CBD (7-OH-CBD), followed by further oxidation and conjugation toward 7-carboxy-CBD (7-COOH-CBD), which is pharmacologically inactive in clinical contexts [2]. In the first-in-human Phase I program, 7-OH-CBD and 7-COOH-CBD were the principal circulating metabolites detected alongside parent CBD, reinforcing the need to interpret both parent and active metabolite in exposure–response assessments [11]. CBD metabolism is primarily dominated by CYP2C and CYP3A families, as demonstrated in studies with human microsomes and recombinant systems examining related CBDs. This evidence supports a mechanistic focus on these isoforms when considering interindividual variability and potential drug interactions involving CBD [36,39]. Moreover, CBD’s high lipophilicity, variable absorption, and propensity for formulation-dependent exposure further highlight the need for concentration-based approaches to personalization rather than reliance solely on administered dose [2,40].

### 4.2. Measuring CBD and Its Metabolites: Bioanalytical Methodology

Validated liquid chromatography–tandem mass spectrometry (LC-MS)/MS methods enable simultaneous quantification of CBD and 7-OH-CBD with clinical laboratory performance characteristics suitable for routine TDM, including defined measuring ranges, acceptable precision, and lower limits of quantification [2]. In Phase I assays, LC-MS achieved minimal matrix effects and inter-assay imprecision compatible with bioanalytical guidance; it demonstrated consistent performance across low-micromolar concentrations—levels typically seen with therapeutic dosing [38]. Complementarily, changes in CYP2C/3A activity can substantially alter the balance between CBD and its metabolites. This reinforces the need for analytical panels that include both the parent compound and 7-OH-CBD, especially when evaluating drug interactions or changes in metabolic capacity [36]. For routine TDM, serum or plasma should be collected as a pre-dose (trough) sample at steady state; as a rule of thumb, steady state is reached after approximately five elimination half-lives (≈5–12 days for CBD given a terminal t½ of ~24–60 h) [26,41,42].

### 4.3. Candidate Reference Ranges and Clinical Interpretation

Data from patients with refractory epilepsy suggest preliminary reference ranges of 0.15–0.50 µmol/L for CBD and 0.04–0.25 µmol/L for 7-OH-CBD. These ranges cover a broad spectrum of maintenance-phase concentrations and offer practical targets for dose adjustment and safety monitoring [2]. These proposals are supported by method validation with measuring ranges spanning sub-micromolar to low-micromolar concentrations, ensuring analytical robustness across subtherapeutic to supratherapeutic concentrations. Phase I studies provide complementary evidence that 7-COOH-CBD, while abundant, is pharmacologically inactive and therefore less relevant for dose optimizations. This supports prioritizing CBD and 7-OH-CBD as the primary analytes in routine reporting [2,38]. Concurrently, classic antiseizure TDM guidance emphasizes that reference intervals must be interpreted alongside clinical response and adverse effects, rather than as rigid therapeutic windows [41].

### 4.4. Variability, Sampling Strategy, and Pre-Analytical Considerations

Real-world TDM data demonstrate pronounced inter- and intra-patient variability in CBD and 7-OH-CBD at comparable daily doses, with only modest dose–concentration correlations, underscoring the limits of dose-based prediction and the value of direct concentration measurement [2]. Accordingly standard TDM principles apply: sampling at steady state, collecting predose (trough) specimens when possible, and repeating measurements during dose titration or following changes in co-medication, organ function, or formulation [41]. After any change in dose, formulation, or interacting medication, trough concentrations should be reassessed once a new steady state is expected (typically within 5–12 days), with feeding state and formulation documented; co-measurement of 7-hydroxy-CBD is recommended to aid interpretation [2,41,42]. Given CBD’s high protein binding and variable absorption, identical doses can yield disparate exposures across patients, making serial concentration-to-dose (C/D) ratios useful to track within-patient trends over time. Although formulation and delivery innovations aim to improve absorption, variability between patients remains significant—making concentration-guided dosing a valuable tool in routine care [2,40].

### 4.5. Drug–Drug Interactions, Active Metabolite, and Clinical Decision Making

CBD’s metabolism via CYP2C19 creates potential for interactions with other drugs processed by the same pathway—most notably benzodiazepines. For example, CBD can significantly increase the concentration ratio of desmethylclobazam to clobazam, which may enhance sedation. However, this effect varies widely between individuals, making lab monitoring essential to balance efficacy with tolerability [2]. When CBD and 7-OH-CBD concentrations fall below the proposed reference intervals with ongoing seizures, we up-titrate in small weekly steps (e.g., by ~5 mg/kg/day) within labeled ranges—typically 10–20 mg/kg/day for LGS/DS and up to 25 mg/kg/day for TSC—and re-evaluate at the next steady state; concentrations above range with adverse effects prompt dose reduction or optimization of sensitive co-therapies (e.g., clobazam dose reduction) [2,5,7,8]. Phase I studies provide a solid foundation for understanding these interactions, including the pharmacokinetics of CBD and its metabolites, assay performance, and protein binding characteristics. Because CYP2C and CYP3A enzymes broadly influence CBD oxidation, insights from microsomal studies support monitoring for enzyme inhibition or induction when treatment regimens change [36,39,42]. CBD is generally well-tolerated, but dose adjustments may be necessary to optimize therapeutic effects and minimize adverse effects, especially in patients receiving multiple medications. Tolerance may develop over time, though this is still under investigation [6]. Finally, CBD TDM should be integrated into broader antiseizure drug monitoring strategies—linking plasma levels to seizure control, liver enzyme changes (e.g., ALT elevations), and adherence—rather than treated as a purely numerical target [2,41] (Table 1).

## 5. Drug–Drug Interactions with Antiseizure Medications

### 5.1. How CBD Interacts with Common Pathways

CBD displays a broad interaction profile, driven primarily because of its reversible inhibition of CYP2C19 and, to a lesser extent, CYP3A4 and several UGT enzymes (UGT1A9, UGT2B7). It also affects drug transporters such as P-glycoprotein; consequently, medications cleared through these pathways may show clinically relevant changes in exposure when co-administered with CBD [26,33,43]. These interactions are further modulated by CBD’s formulation-dependent bioavailability, the impact of food on absorption, and interpatient pharmacokinetic variability, all of which strengthen the case for concentration-guided management in practice [2,27]. Mechanistic cross-talk at 5-HT1A, adenosinergic, and GABAergic nodes can create pharmacodynamic synergy—beneficial or adverse—when CBD is combined with sedative ASMs [9,10] (Table 2).

### 5.2. Key Drug Interactions

#### 5.2.1. Clobazam

The most robust CBD–ASM pharmacokinetic interaction involves clobazam (CLB): CBD inhibits CYP2C19, producing a several-fold rise in N-desmethylclobazam (N-CLB), with a lesser effect on CLB itself; somnolence and sedation correlate with this metabolite accumulation and typically improve after CLB dose reduction [1,33,43]. Meta-analyses and stratified reads of trial data indicate that CBD retains antiseizure activity independent of CLB, although effect sizes are generally larger with the combination; this duality explains differing regulatory labels and supports proactive N-CLB monitoring in sedated patients [44]. Real-world series confirm responders both with and without CLB, but benzodiazepine co-therapy increases the risk of dose-limiting drowsiness, guiding a practical sequence of titrating CBD while pre-emptively down-titrating CLB by 25–50% if sedation emerges [45,46]. In the broader LGS therapeutic armamentarium, expert panels position CBD alongside other tier 1 options, further normalizing CLB-aware co-prescription strategies [47].

#### 5.2.2. Valproate

Controlled studies show CBD does not substantially alter valproate (VPA) exposure, yet the combination produces dose-related transaminase elevations, implicating a mechanism other than simple displacement kinetics; baseline and early on-therapy liver function testing are therefore essential [1,43,48]. When hepatotoxicity risk factors coexist, such as polytherapy or pre-existing hepatic vulnerability, slower CBD up-titration and more frequent laboratory monitoring are prudent [13,27].

#### 5.2.3. Stiripentol

Stiripentol (STP), a potent CYP2C19 inhibitor, can augment the CBD-associated rise in N-CLB and occasionally modify CBD or STP exposures; although the net pharmacokinetic shifts are usually small, vigilance for additive sedation in triple therapy (CBD+CLB+STP) is warranted [33,37,43]. In practice, splitting CBD doses and performing an early clinical review after STP introduction helps prevent excessive benzodiazepine-related adverse effects [22].

### 5.3. Other Potential Interactions

CBD can increase concentrations of several co-administered ASMs—most consistently brivaracetam, and variably topiramate, zonisamide, and rufinamide—changes that are generally modest yet can occasionally exceed beyond typical reference ranges; targeted level checks are reasonable when clinical response or tolerability deviates from expectation [2,43]. Other potential interactions of CBD antiseizure drugs primarily involve hepatic cytochrome P450 enzymes. For example, CBD can elevate lamotrigine levels by inhibiting UGT2B7, while drugs like carbamazepine, oxcarbazepine, and phenobarbital reduce CBD levels through CYP3A4 induction [9]. Levetiracetam shows no consistent pharmacokinetic interaction, though preclinical data suggest context-dependent pharmacodynamic interplay, underscoring the value of symptom-driven dose refinements rather than anticipatory adjustments [9,43]. Outside the ASM class, exposure increases in mTOR inhibitors (sirolimus/everolimus) have been repeatedly observed, a reminder that CBD’s inhibitory profile extends to oncology and immunology co-therapies often encountered TSC [2,43]. Given CBD’s central nervous system polypharmacy footprint (5-HT1A, adenosine, TRP, GABA), additive sedation with benzodiazepines and other central depressants is mechanistically credible and empirically common [10,49]. A practical clinical strategy that has proven serviceable across trials and observational cohorts includes maximizing non-sedating ASMs when feasible, introducing CBD with food at a stable dose, checking trough concentrations or surrogate TDM for CBD/7-OH-CBD and sensitive co-medications when adverse effects or loss of efficacy occur, and first adjusting the agent with the narrowest therapeutic index [1,2,13,22,27,36].

## 6. Clinical Applications in Epilepsy Syndromes

### 6.1. Lennox–Gastaut Syndrome (LGS)

In LGS, adjunctive highly purified CBD produces clinically relevant reductions in drop seizures and delivers benefits that extend beyond seizure counts to patient-centered outcomes [50,51,52]. In phase-3 trials (GWPCARE3/4), median reductions in drop-seizure frequency were approximately 37–42% with CBD versus approximately 17% with placebo, with parallel improvements across non-drop seizure types, establishing consistent efficacy across the LGS seizure spectrum [22,53]. Building on these trials, a pooled post hoc analysis introduced seizure-free days (SFDs) as a complementary endpoint, showing least-squares mean differences versus placebo of approximately 2.8–3.6 additional SFDs per 28 days during treatment and maintenance periods, thereby quantifying day-to-day benefit perceived by patients and caregivers [50]. Caregiver interviews from a multinational qualitative study reported enhancements in communication, behavior, mobility, and family routines during CBD therapy, suggesting that seizure control translates into broader participation and quality-of-life gains [51]. Real-world data from Korea corroborate feasibility and effectiveness in routine multidisciplinary programs, with initiation at 5 mg/kg/day and maintenance near 10 mg/kg/day yielding meaningful seizure reductions without life-threatening events [54].

Dose selection in LGS reflects both trial designs and emerging patient-reported anchors of meaningful change [52,53]. Although 10–20 mg/kg/day remains the standard adjunctive range evaluated in the pivotal studies, exploratory analyses anchored to the Clinical Global Impression–Change (CGI-C) scale—a clinician-rated global improvement metric—indicate that an approximately 31% reduction in drop seizures may represent a clinically important response for many families, informing shared decision-making when classical ≥50% responder thresholds are not met [52]. Within syndrome-specific care pathways, CBD is typically integrated after first-line options (e.g., valproate) and used alongside nonpharmacologic supports, with practical attention to titration cadence and adverse-event surveillance appropriate to the LGS comorbidity profile [53,55]. The adverse-effect profile in LGS trials is predictable—somnolence, decreased appetite, and diarrhea—with transaminase elevations occurring more often under valproate co-therapy; programmatic liver function testing and symptom-triggered adjustments help to preserve benefit across maintenance phases [7,22,48]. Collectively, these LGS-focused data emphasize outcomes that matter in daily life—additional seizure-free days, caregiver-perceived improvement, and thresholds of “clinically meaningful” reduction—complementing conventional responder analyses and informing individualized goals of therapy [50,51,52].

### 6.2. Dravet Syndrome

Under contemporary standards of care, adjunctive highly purified CBD produces clinically meaningful reductions in convulsive—seizure frequency among patients with DS. In randomized controlled trials reporting approximately 43% for CBD-treated participants achieved at least a 50% reduction in seizure frequency, compared with approximately 27% receiving placebo, both on stable background therapy. Dose-ranging studies show comparable efficacy at 10 and 20 mg/kg/day, supporting the use of the lower dose to optimize tolerability [28,56]. Within modern DS treatment algorithms, CBD is positioned alongside stiripentol and fenfluramine as a licensed option, and synthesis papers emphasize that benefits extend beyond a single seizure phenotype into caregiver-relevant domains such as sleep, irritability, and daily functioning, typically captured as secondary or exploratory endpoints [56,57]. Real-world programs complement these trials: in the UK Early Access Program, maintenance doses often stabilized below pivotal-trial maxima (approximately 6–7 mg/kg/day at 6–12 months) while reductions in convulsive seizures remained clinically meaningful, supporting individualized maintenance dosing guided by tolerability and observed benefit [56,58]. Importantly, although pivotal randomized controlled trials evaluated 10–20 mg/kg/day, open-label extension cohorts permitted higher targets—up to 25–50 mg/kg/day under specialist supervision—indicating that doses above 20 mg/kg/day are feasible in selected cases when incremental benefit justifies closer monitoring [28,56]. Interaction-aware practice is essential in DS: CBD inhibits CYP2C19 and can substantially increase N-desmethylclobazam exposure; therefore, clinicians frequently pre-empt or promptly implement clobazam dose reductions (often 25–50%) when somnolence or ataxia emerge; simultaneously, hepatic enzyme elevations occur more often with concomitant valproate, warranting baseline and periodic liver function testing during titration and maintenance [59,60]. Contemporary consensus guidance advocates gradual up-titration—typically starting at 2.5 mg/kg twice daily (5 mg/kg/day), increasing to 10 mg/kg/day within approximately one week, and considering escalation to 20 mg/kg/day in partial responders; a slower titration schedule is advised when patients are on polytherapy or have a higher risk of adverse effects [1,28,60]. When TDM is used to navigate interindividual variability typical of DS polytherapy, obtaining trough samples at steady state and concurrently measuring 7-hydroxy-CBD can help distinguish nonadherence from true underexposure and interpret interaction-driven concentration changes. Accurate interpretation depends on laboratories reporting the sample matrix, assay parameters, and interpretive ranges. Overall, contemporary DS treatment frameworks position CBD as a key, interaction-aware adjunct aimed at optimizing convulsive seizure control and improving patient-centered outcomes within a structured, safety-focused care model [57,61].

### 6.3. Tuberous Sclerosis Complex (TSC)

In TSC, adjunctive highly purified CBD confers clinically meaningful reductions in total seizure frequency across multiple seizure types, with the pivotal randomized controlled trial demonstrating median reductions of approximately 49% and 48% at 25 and 50 mg/kg/day, respectively, versus approximately 27% with placebo, and showing that the lower dose achieves similar efficacy with fewer adverse events [62]. Current European recommendations position CBD as a licensed adjunct for TSC—associated seizures (in patients aged ≥2 years), advising typical maintenance up to 25 mg/kg/day and emphasizing vigilance for drug–drug interactions when CBD is incorporated into TSC care pathways [63]. CBD use should be considered within the context of disease-modifying regimens that frequently include the mTOR inhibitor everolimus. Clinical guidance highlights an “important drug–drug interaction” between these agents and recommends appropriate laboratory surveillance; this reflects everolimus’ well-established role in early TSC management and its independent antiseizure efficacy [63,64]. In practice, these data support initiating CBD with a tolerability-focused strategy that favors the lower effective dose range, incorporates baseline and periodic liver function testing, and reviews concomitant CYP3A4 substrates/inducers. When CBD is co-administered with mTOR inhibitors, trough monitoring of everolimus and corresponding dose adjustment should be considered as clinically indicated [62,63].

### 6.4. Other Neurological and Systemic Indications

#### 6.4.1. Neurodevelopmental and Psychiatric Conditions

Early clinical signals suggest that CBD may attenuate anxiety and agitation phenotypes, with acute anxiolytic effects demonstrated in simulated public speaking paradigms at 300–600 mg in adults and mechanistic links proposed via 5-HT1A, adenosinergic, and limbic network modulation; however, optimal dosing and durability remain unsettled, and sedation can emerge at higher doses [65,66]. Emerging psychosis studies indicate that antipsychotic-like effects typically require 800–1000 mg/day, yet results are mixed and call for standardized trials to clarify benefit–risk and patient selection criteria [66]. In addition, early studies in autism spectrum disorder report reductions in irritability/agitation and caregiver-observed behavioral improvements with purified CBD, although dosing durability and standardized outcome frameworks remain to be established [66].

#### 6.4.2. Movement and Sleep Disorders

Pilot work in Parkinson’s disease and related sleep–motor phenotypes report heterogeneous outcomes, with small trials and case series suggesting improvements in certain non-motor domains and REM sleep behavior disorder. However, consistent effect sizes and dose–response relationships are not yet established; mechanistic plausibility (e.g., 5-HT1A and TRP channel signaling) supports further phase II testing. Exploratory case series in dystonia suggest context-dependent symptom modulation with CBD, but controlled trials are lacking and dose–response relationships remain undefined [66,67].

#### 6.4.3. Pain and Spasticity

For chronic pain conditions, controlled evidence for isolated oral CBD remains sparse and often inconclusive, in contrast to tetrahydrocannabinol (THC)/CBD combinations (e.g., nabiximols) that predominate positive trials; existing CBD studies show variable designs, routes (including transdermal), and doses (tens to low hundreds of mg/day), underscoring a need for placebo-controlled, dose-ranging trials before routine clinical adoption. For multiple sclerosis spasticity, positive signals derive primarily from THC/CBD combinations rather than isolated CBD, underscoring the translational limitation for pure-CBD practice [66,68].

#### 6.4.4. Inflammatory, Dermatologic, and Gastrointestinal Indications

Translation from robust preclinical anti-inflammatory and neuromodulatory actions to human benefit in inflammatory bowel disease, arthritic dermatoses, and related conditions remains preliminary, constrained by small, heterogeneous studies and product-quality variability; consequently, dosing remains non-standardized and any off-label use should be confined to research settings with assay-verified preparations [66,67].

## 7. Future Directions

Priorities for the next phase of CBD therapeutics include defining long-term benefit–risk across the lifespan and converting PK variability into precision dosing algorithms linked to patient-centered outcomes [33,61,69]. Real-world and extension cohort data suggest that sustained use of CBD is generally well-tolerated with reasonable retention rates. However, pediatric neurodevelopmental outcomes, sleep parameters, and quality-of-life trajectories remain insufficiently characterized and should be incorporated prospectively into long-term studies [69,70,71]. Optimizing bioavailability is a practical strategy: In a Phase I trial in healthy volunteers, oral CBD formulations (oil solution and sublingual wafer) were generally well tolerated and produced dose-dependent increases in systemic exposure, with a terminal half-life in the range of several hours [42]. Advances in formulation science, including nano-enabled oral delivery systems, have shown incremental improvements over traditional oromucosal routes and warrant comparative studies against the approved oral solution using PK/pharmacodynamics (PD) endpoints [33]. Given that hepatotoxicity risks increase with polytherapy, there is a need for targeted research investigating mechanistic links between reactive metabolites and liver injury signals, alongside feasible transaminase monitoring strategies suitable for real-world settings [33,70].

Integrating pharmacogenomics with TDM offers the potential to minimize avoidable exposure variability. CYP2C19 polymorphisms, common especially in Asian populations, support genotype-guided dose titration and interpretation frameworks that include both CBD and its active metabolite 7-OH-CBD measured at steady-state trough levels [2,33]. Harmonized laboratory reporting encompassing sampling conditions, assay parameters, and decision thresholds should align with best-practice TDM standards while accommodating CBD-specific assay and matrix considerations [2,41]. Beyond epileptic encephalopathies, emerging priority indications include neurodegenerative disorders, circuit-level modulation captured by human neuroimaging, and psychiatric conditions—areas where dosing bands, exposure–response relationships, and safety margins for pure CBD remain to be formalized [20,65,72]. In genetic epilepsies such as CDKL5 deficiency disorder, there are opportunities to combine model-informed dosing approaches with syndrome-specific clinical endpoints [73]. Regulatory standardization and product quality control remain crucial, as discrepancies between GMP-grade, highly purified CBD products and “CBD-enriched” oils widely used in some adult treatment series complicate data comparability. Addressing this through assay traceability, content uniformity standards, and bridging studies is paramount [33,74]. Dose-finding research comparing mg/kg/day tiers—ideally integrating fed-state dosing, pharmacogenomics, TDM, and patient-reported outcome measures—can guide the selection of the lowest effective maintenance dose across diverse adult and pediatric populations [20,42,70]. An essential area for future research is investigating CBD’s interaction with Nav1.7 and Nav1.2 channels, particularly in drug-resistant epilepsy. Recent studies have shown that CBD binds to the inactivated state of Nav1.7 channels, stabilizing them and potentially offering a novel therapeutic target. Additionally, CBD selectively inhibits resurgent currents in Nav1.2 channels, a feature that may be leveraged to treat refractory epilepsy with fewer side effects compared to conventional ASMs [75,76].

## 8. Conclusions

CBD acts as a multitarget antiseizure agent with pharmacology largely independent of CB1/CB2 receptors, focusing instead on GPR55 antagonism, TRP-channel desensitization, and adenosine-mediated neural network modulation. Clinically, its performance is governed by a distinct pharmacokinetic profile characterized by low oral bioavailability, a pronounced food effect, extensive tissue distribution, and phase I/II metabolism that produces the active 7-hydroxy-CBD and the abundant but inactive 7-carboxy-CBD. These features, together with critical interaction liabilities—such as CYP2C19-mediated elevation of N-desmethylclobazam, liver enzyme elevations linked to valproate, and fluctuations from CYP-inducing or inhibiting co-medications—make individualized treatment essential. Evidence-based dosing should be coupled with liver function monitoring, interaction-aware titration, and therapeutic drug monitoring of CBD and 7-OH-CBD at steady-state trough concentrations to distinguish underexposure from nonadherence and to standardize administration relative to fed state and formulation. Looking forward, advances in pharmacogenomics integration, bioavailability optimization through novel delivery, and tighter analytical and product quality controls will establish CBD as a precision-ready cornerstone of contemporary epilepsy care.

## Figures and Tables

**Table 1:** Therapeutic Drug Monitoring of Cannabidiol: Analytes, Sampling, Reference Intervals, and Actionable Decision Rules.

Domain	CBD (Parent)	7-OH-CBD (Active Metabolite)
**Analyte priority**	Core target for level-guided dosing	Core; interpret alongside CBD
**Matrix/method**	Serum or plasma; validated LC-MS/MS with reported LLOQ and precision	Same specimen/method as CBD
**Sampling time**	Pre-dose (trough) at steady state	Same timing (paired with CBD)
**When to recheck**	After any change in dose, formulation, or interacting drug, re-assess once a new steady state is expected (~5–12 days)	Same
**Pre-analytical documentation**	Feeding state (high-fat or standard), formulation (oral solution vs. others), clock time of last dose, total daily dose, co-medications, ALT/AST, adherence notes	Same
**Proposed reference interval**	0.15–0.50 μmol/L	0.04–0.25 μmol/L
**Factors that increase levels**	High-fat meals; CYP inhibitors; hepatic impairment; reduced CYP2C19 activity	Often rises in parallel with CBD; may be accentuated with greater metabolic conversion
**Factors that decrease levels**	CYP inducers; fasted sampling; switch to lower-bioavailability formulation; missed doses	Typically tracks with CBD decreases
**Interpretive patterns**	CBD low & 7-OH-CBD low: underexposure or nonadherence/fasted state. CBD in range & 7-OH-CBD high: enhanced metabolism or timing error. CBD high ± adverse effects: review feeding/formulation and interactions.	Use the ratio to contextualize patterns above
**Actionable decision rules**	Below range with seizures: up-titrate in small weekly steps (~5 mg/kg/day) within labeled ranges (10–20 mg/kg/day for LGS/DS; up to 25 mg/kg/day for TSC), then re-check at the next steady state. Above range with adverse effects: reduce dose or optimize sensitive co-therapies (e.g., clobazam dose reduction); within range: maintain and continue LFT surveillance.	Interpret changes together with CBD; disproportionate 7-OH-CBD suggests metabolism/interaction effects that may guide co-therapy adjustments

Note: parent = unchanged parent compound measured in plasma/serum. This table operationalizes CBD TDM by specifying (i) the analytes to measure (CBD and 7-OH-CBD), (ii) standardized trough sampling at steady state, and (iii) paired interpretation using proposed reference intervals. Because CBD exposure varies with feeding state and formulation, these conditions should be held constant and documented at each draw; re-assessment is recommended once a new steady state is expected (~5–12 days) after any dose, formulation, or interaction change. Dose adjustments are implemented as small weekly steps (~5 mg/kg/day) within labeled ranges (10–20 mg/kg/day for LGS/DS; up to 25 mg/kg/day for TSC), while elevations with adverse effects prompt dose reduction or interaction-aware optimization (e.g., clobazam down-titration) and continued LFT surveillance. Using paired CBD/7-OH-CBD results helps distinguish underexposure or nonadherence from interaction-driven shifts and supports individualized maintenance dosing. Abbreviations: CBD, cannabidiol; 7-OH-CBD, 7-hydroxy-cannabidiol; LGS, Lennox–Gastaut syndrome; DS, Dravet syndrome; TSC, tuberous sclerosis complex; LC-MS/MS, liquid chromatography–tandem mass spectrometry; LLOQ, lower limit of quantification; ALT/AST, alanine/aspartate aminotransferase (liver function tests).

**Table 2:** Clinically Relevant Drug–Drug Interactions Between Cannabidiol and Antiseizure Medications: Mechanisms, Magnitude, Consequences, and Management.

ASM (Co-Therapy)	Predominant Mechanism	Direction/Magnitude	Clinical Consequence	Recommended Management
**Clobazam (CLB)**	CYP2C19 inhibition by CBD, with NCLB ↑	Increase, several-fold	Somnolence/sedation, ataxia; efficacy amplified	Pre-empt/early CLB reduction (25–50%); consider N-CLB check; caregiver education
**Valproate (VPA)**	PD/hepatic signal (no consistent PK shift)	Transaminases ↑ (dose-related)	ALT/AST elevations more frequent	Baseline & periodic LFTs; slower CBD up-titration; re-check after changes
**Stiripentol (STP)**	CYP2C19 inhibitor; interacts with CLB pathway	Augments N-CLB rise; small CBD/STP shifts	Additive sedation in CBD+CLB+STP	Split CBD dose; early review; adjust CLB first if sedation
**Brivaracetam (BRV)**	Not fully defined; possible metabolic interplay	Modest ↑	Occasional overshoot of reference range	Targeted level check if AEs/efficacy change; symptom-driven titration
**Topiramate (TPM)**	Unknown/indirect	Variable, modest ↑	Headache/appetite/behavioral AEs	Check if off-trajectory; clinical monitoring
**Zonisamide (ZNS)**	Unknown/indirect	Variable, modest ↑	Cognitive/behavioral AEs possible	Monitor and adjust if needed
**Rufinamide (RUF)**	Unknown/indirect	Variable, modest ↑	Somnolence/dizziness may increase	Consider dose refinement if AEs emerge
**Levetiracetam (LEV)**	No consistent PK interaction	Neutral (PK); possible PD interplay	Irritability/behavior may fluctuate	Symptom-driven adjustments; no routine PK change
**Everolimus/Sirolimus (TSC context)**	CYP3A4/P-gp substrates; CBD inhibitory profile	Exposure ↑ (clinically relevant)	Mucositis, hyperlipidemia risk ↑	Trough-guided dose adjustment; document start/stop timing

This table synthesizes documented interactions between highly purified cannabidiol (CBD) and commonly co-administered ASMs (and selected co-therapies). The dominant mechanism is reversible CYP2C19 inhibition by CBD (with additional effects at CYP3A4 and UGT1A9/UGT2B7), which increases N-desmethylclobazam exposure and sedation, often requiring a 25–50% clobazam dose reduction. Valproate shows no consistent PK shift but is associated with dose-related transaminase elevations, mandating baseline and periodic LFT monitoring; stiripentol may further augment N-CLB in triple therapy (CBD+CLB+STP). Brivaracetam and, variably, topiramate/zonisamide/rufinamide can display modest concentration increases, whereas levetiracetam has no consistent PK interaction (possible PD effects); in TSC care, everolimus/sirolimus exposures may increase and benefit from trough-guided dose adjustment. Management emphasizes interaction-aware titration, pre-emptive clobazam reduction when sedation emerges, LFT surveillance with valproate, and selective TDM (CBD and 7-OH-CBD) when efficacy or tolerability deviates from expectation. Symbols: ↑, increase. Abbreviations: ASM, antiseizure medication; CBD, cannabidiol; CLB, clobazam; N-CLB, N-desmethylclobazam; VPA, valproate; LFT(s), liver function test(s); STP, stiripentol; BRV, brivaracetam; TPM, topiramate; ZNS, zonisamide; RUF, rufinamide; LEV, levetiracetam; PK, pharmacokinetic; PD, pharmacodynamic; CYP, cytochrome P450; UGT, UDP-glucuronosyltransferase; P-gp, P-glycoprotein; TSC, tuberous sclerosis complex; TDM, therapeutic drug monitoring; 7-OH-CBD, 7-hydroxy-cannabidiol; AE(s), adverse event(s).

## Data Availability

No new data were created or analyzed in this study. Data sharing is not applicable to this article.

## Abbreviations

The following abbreviations are used in this manuscript:

CBD	cannabidiol
THC	tetrahydrocannabinol
LGS	Lennox–Gastaut syndrome
DS	Dravet syndrome
TSC	tuberous sclerosis complex
PK	pharmacokinetics
TDM	therapeutic drug monitoring
GPR55	G protein-coupled receptor 55
TRP	transient receptor potential
CYP2C19	cytochrome P450 2C19
CYP3A4	cytochrome P450 3A4
UGT	uridine 5′-diphospho-glucuronosyltransferase
7-OH-CBD	7-hydroxy-cannabidiol
7-COOH-CBD	7-carboxy-cannabidiol
ASM	antiseizure medication
CLB	clobazam
N-CLB	N-desmethylclobazam
VPA	valproate
STP	stiripentol
PK/PD	pharmacokinetic/pharmacodynamic
ALT	alanine aminotransferase
SFD	seizure-free days
CGI-C	Clinical Global Impression–change
ENT1	equilibrative nucleoside transporter 1
VDAC	voltage-dependent anion channel
